# Poor Glycemic Control of Diabetes Mellitus Is Associated with Higher Risk of Prostate Cancer Detection in a Biopsy Population

**DOI:** 10.1371/journal.pone.0104789

**Published:** 2014-09-08

**Authors:** Juhyun Park, Sung Yong Cho, Young Ju Lee, Seung Bae Lee, Hwancheol Son, Hyeon Jeong

**Affiliations:** 1 Department of Urology, Seoul Metropolitan Government Seoul National University Boramae Medical Center, Dongjak-gu, Seoul, Korea; 2 Department of Urology, Seoul National University Hospital 101, Daehak-Ro Jongno-gu, Seoul, Korea; Innsbruck Medical University, Austria

## Abstract

**Objectives:**

To evaluate the impact of glycemic control of diabetes mellitus (DM) on prostate cancer detection in a biopsy population.

**Patients and Methods:**

We retrospectively reviewed the records of 1,368 men who underwent prostate biopsy at our institution. We divided our biopsy population into three groups according to their history of DM, and their Hemoglobin A1c (HbA1c) level: a no-DM (DM−) group; a good glycemic control (DM+GC) group (HbA1c <6.5%); and a poor glycemic control (DM+PC) group (HbA1c ≥6.5%). For sub-analyses, the DM+PC group was divided into a moderately poor glycemic control (DM+mPC) group (6.5≤ HbA1c <7.5%) and a severely poor glycemic control (DM+sPC) group (HbA1c ≥7.5%).

**Results:**

Among 1,368 men, 338 (24.7%) had a history of DM, and 393 (28.7%) had a positive biopsy. There was a significant difference in prostatic specific antigen density (PSAD) (P = 0.037) and the frequency of abnormal DRE findings (P = 0.031) among three groups. The occurrence rate of overall prostate cancer (P<0.001) and high-grade prostate cancer (P = 0.016) also presented with a significantly difference. In the multivariate analysis, the DM+PC group was significantly associated with a higher rate of overall prostate cancer detection in biopsy subjects compared to the DM− group (OR = 2.313, P = 0.001) but the DM+PC group was not associated with a higher rate of high-grade (Gleason score ≥7) diseases detected during the biopsy (OR = 1.297, P = 0.376). However, in subgroup analysis, DM+sPC group was significantly related to a higher risk of high-grade diseases compared to the DM− group (OR = 2.446, P = 0.048).

**Conclusions:**

Poor glycemic control of DM was associated with a higher risk of prostate cancer detection, including high-grade disease, in the biopsy population.

## Introduction

The hypothesis that diabetes mellitus (DM) and prostate cancer have an inverse relationship was accepted as a medical fact quite recently. Especially, two meta-analysis papers published in 2004 and 2006 strongly supported this contention [1], [2]. However, Chan JM and his colleagues announced that they could not find any evidence of the inverse relationship between DM and prostate cancer [3]. Some papers suggested that the relationship between DM and high-grade prostate cancer changed according to the activity of the patient, their body mass index (BMI), and ethnicity [4]–[6].

Several Asian papers also refuted the former hypothesis. Hong et al. concluded that DM was associated with a higher risk of detection of prostate cancer in a Korean population [7]. Two Japanese studies reported that DM was associated with aggressive or advanced prostate cancer [8], [9].

Meanwhile, some studies researched the influence of DM by measuring hemoglobin A1c (HbA1c) level. To understand the effect of DM on prostate cancer, analyzing the difference according to the degree of glycemic control is considered as more appropriate rather than merely checking for the existence of a DM history. However, the studies about these subjects are lacking and the results have been controversial [10]–[14].

Consequently, we investigated the potential effect of DM according to the glycemic control level on prostate cancer detection in a biopsy population by using HbA1c measurements.

## Materials and Methods

After institutional review board approval, we retrospectively reviewed the medical records of patients who underwent initial transrectal ultrasound-guided 12-core prostate biopsy at our clinic between January 2008 and October 2013. Indications for prostate biopsy were a serum prostate-specific antigen (PSA) level ≥4 ng/ml or a positive digital rectal examination (DRE) performed by urologists for all patients. Patients with a history of a previous biopsy at another institution, surgical treatment for prostatic disease, or incomplete clinical data were excluded from our study. An 18-gauge core biopsy needle and automatic spring-loaded biopsy gun were used. Systematic 12-core biopsies were performed at the apices, midportions, bases, and peripheral area of both planes. If patients had a target lesion (i.e., a hypoechoic lesion), and the site of the lesion was not included in the routine systematic 12-core biopsy sites, the uroradiologists conducted an additional biopsy of the target lesion.

Patient age, PSA level, DRE findings, DM history, HbA1c level, and prostate volume and the biopsy Gleason sum were analyzed to assess the potential association between the glycemic control of DM and prostate cancer detection. In addition, the effect on the detection of high-grade (Gleason ≥7) prostate cancer was analyzed.

The DM history was determined by primary physicians or internal medicine doctors. We checked HbA1c only if the patients had a history of DM, which we determined by taking their medical histories. We divided our biopsy population into three groups according to their history of DM, and HbA1c level: a no-DM (DM−) group; a good glycemic control (DM+GC) group (HbA1c <6.5%); and a poor glycemic control (DM+PC) group (HbA1c ≥6.5%). For subgroup analyses, the DM+PC group was divided into a moderately poor glycemic control (DM+mPC) group (6.5≤ HbA1c <7.5%) and a severely poor glycemic control (DM+sPC) group (HbA1c ≥7.5%). Additionally, a subgroup analysis for age was assessed by decades, the PSA analysis was based on a cut-off level of 10 ng/ml. Obesity was defined as a BMI ≥25 kg/m^2^.

We used Pearson's Chi-square test, Fisher's exact test, and the linear regression model to describe the relationship between the variables. Multivariate logistic and linear regression analysis was applied to examine the association between the glycemic control of DM and the prostate cancer on biopsy, adjusting for age, prostate volume, PSA level, and DRE findings. The association with the detection of high-grade cancer was similarly evaluated. Statistical significance was considered as P<0.05. All statistical analyses were performed using commercially available software (SPSS 14.0, Chicago, IL, USA).

## Results

A total of 1,368 patients were included in the analysis, the mean age was 66.7 years and the median PSA level was 6.9 ng/ml. There were 338 men (24.7%) with a history of DM. Prostate cancer was detected from the biopsy in 393 (28.7%) patients and high-grade (Gleason score ≥7) prostate cancer was found in 263 (19.2%) (Table 1).

**Table 1:** Patient characteristics.

Parameter	
No. Patient	1,368
Mean Age (range)	66.7 (30–91)
Median PSA (ng/ml, range)	6.9 (1.0–3081.0)
Median PSAD (ng/ml^2^, range)	0.17 (0.03–13.32)
No. abnormal DRE finding (%)	176 (12.9%)
Mean prostate volume (ml, range)	45.0 (9–262)
No. DM (%)	338 (24.7%)
No. overall Pca (%)	393 (28.7%)
No. high-grade Pca (GS ≥7) (%)	263 (19.2%)
GS 7	131 (9.6%)
GS 3+4	76 (5.6%)
GS 4+3	55 (4.0%)
GS 8	73 (5.3%)
GS 9	18 (1.3%)
GS 10	12 (0.9%)

PSA, prostate-specific antigen; PSAD, prostate-specific antigen density; DRE, digital rectal examination; DM, diabetes mellitus; Pca, prostate cancer; GS, Gleason Score.

Table 2 shows the comparison among three groups by the degree of glycemic control based on the HbA1c level and the history of DM (DM−, DM+GC, DM+PC). There was a significant difference in prostatic specific antigen density (PSAD) (P = 0.037) and the frequency of abnormal DRE findings (P = 0.031). The occurrence rate of overall prostate cancer was significantly different (P<0.001) and high-grade prostate cancer presented with a significantly different occurrence rate (P = 0.016).

**Table 2:** Patient characteristics and biopsy outcomes according to glycemic control of DM.

Variables	DM−	DM+GC	DM+PC	P-value
No. Patient	1,030	238	100	
Mean Age (range)	66.7 (30–91)	66.0 (34–86)	67.8 (47–83)	0.210
Median PSA (ng/ml, range)	7.2 (1.4–3081.0)	6.0 (1.0–412.0)	6.1 (1.2–42.1)	0.207
Median PSAD (ng/ml^2^, range)	0.18 (0.03–13.32)	0.15 (0.03–1.37)	0.14 (0.04–0.72)	0.037
No. abnormal DRE finding (%)	128 (12.4%)	27 (11.3%)	21 (21.0%)	0.031
Mean prostate volume (ml, range)	45.0 (9–262)	44.5 (14–126)	45.5 (18–151)	0.949
Mean HbA1c (range)	-	5.7 (4.4–6.4)	7.4 (6.5–11.3)	<0.001
No. overall Pca (%)	301 (29.2%)	48 (20.2%)	44 (44.0%)	<0.001
No. high-grade Pca (GS ≥7) (%)	211 (20.5%)	30 (12.6%)	22 (22.0%)	0.016
GS 7	106 (10.3%)	15 (6.3%)	10 (10.0%)	
GS 3+4	61 (5.9%)	8 (3.4%)	7 (7.0%)	
GS 4+3	45 (4.4%)	7 (2.9%)	3 (3.0%)	
GS 8	57 (5.5%)	8 (3.4%)	8 (8..0%)	
GS 9	14 (1.4%)	1 (0.4%)	3 (3.0%)	
GS 10	11 (1.1%)	1 (0.4%)	0 (0%)	

DM, diabetes mellitus, DM−, no DM; DM+GC, DM with good glycemic control, HbA1c <6.5%; DM+PC, DM with poor glycemic control, HbA1c ≥6.5%; PSA, prostate-specific antigen; PSAD, prostate-specific antigen density; DRE, digital rectal examination; Pca, prostate cancer; GS, Gleason Score.

In men with negative biopsies, the mean PSA level was 9.10 ng/ml in the DM− group, 7.52 ng/ml in the DM+GC group, and 7.28 ng/ml in the DM+PC group. These figures showed a significantly difference among the groups (P = 0.015).

The DM+PC group was significantly associated with a higher risk of detection of overall prostate cancer than the DM− group via biopsy in univariate (OR = 1.093, P = 0.002) and multivariate analyses (OR = 2.313, P = 0.001). Meanwhile, the DM+PC group was not associated with a higher rate of high-grade diseases compared to the DM− group in univariate (OR = 1.095, P = 0.721) and multivariate analyses (OR = 1.297 P = 0.376) (Table 3).

**Table 3:** Odds ratio of DM groups being associated with overall prostate cancer or high-grade (Gleason score ≥7) prostate cancer detection on prostate biopsy.

Overall prostate cancer detection				
	**Unadjusted**		**Adjusted**	
	**OR (95% CI)**	**P-value**	**OR (95% CI)**	**P-value**
Age	1.059 (1.043–1.076)	<0.001	1.062 (1.043–1.081)	<0.001
PSA	1.043 (1.030–1.056)	<0.001	1.051 (1.033–1.069)	<0.001
Prostate volume	0.974 (0.966–0.981)	<0.001	0.957 (0.948–0.966)	<0.001
Abnormal DRE finding	2.341 (1.661–3.300)	<0.001	1.556 (1.033–2.345)	0.034
DM−	reference	-	reference	-
DM+GC	0.612 (0.434–0.863)	0.005	0.759 (0.517–1.115)	0.160
DM+PC	1.903 (1.254–2.888)	0.002	2.313 (1.408–3.799)	0.001
**High-grade prostate cancer**				
	**Unadjusted**		**Adjusted**	
	**OR (95% CI)**	**P-value**	**OR (95% CI)**	**P-value**
Age	1.062 (1.043–1.082)	<0.001	1.057 (1.035–1.079)	<0.001
PSA	1.039 (1.027–1.051)	<0.001	1.066 (1.046–1.085)	<0.001
Prostate volume	0.973 (0.965–0.982)	<0.001	0.951 (0.940–0.963)	<0.001
Abnormal DRE finding	1.967 (1.351–2.865)	<0.001	1.270 (0.803–2.008)	0.307
DM−	reference	-	reference	-
DM+GC	0.560 (0.371–0.845)	0.006	0.692 (0.434–1.104)	0.122
DM+PC	1.095 (0.666–1.799)	0.721	1.297 (0.729–2.307)	0.376

OR, odds ratio for positive biopsy; CI, confidence interval; DRE, digital rectal examination, DM, diabetes mellitus; DM−, no DM; DM+GC, DM with good glycemic control, HbA1c <6.5%; DM+PC, DM with poor glycemic control, HbA1c ≥6.5%.

When we divided the DM+PC group for the subgroup analysis, there were 69 men in the DM+mPC group and 31 men in the DM+sPC group. Multivariate analysis showed a significantly risk elevation of overall prostate cancer detection in both of the DM+mPC group (OR = 2.162, P = 0.011) and the DM+sPC group (OR = 2.670, P = 0.022), compared to DM− group. In addition, the DM+sPC group was significantly associated with a higher rate of high-grade prostate cancer detection than the DM− group in the multivariate analysis (OR = 2.444, P = 0.048).

When our analysis was limited to men with PSA <10 ng/ml, the DM+PC group was associated with a higher rate of overall prostate cancer detection compared to the DM− group from the biopsy in multivariate analysis (OR = 2.329 P = 0.003). However, multivariate analysis for high-grade prostate cancer showed no significant findings.

When stratified by age, only for men older than 60 years, the DM+PC group was significantly associated with a higher risk of overall prostate cancer detection than the DM− group in our biopsy population after multivariate analysis (OR = 2.111, P = 0.005), and it also applied for younger men (OR = 5.320, P = 0.016). In multivariate analysis for high-grade prostate cancer, there was no significant association for patients older than 60 years, whereas the DM+PC group showed borderline significance regarding the risk of high-grade prostate cancer detection for younger men (OR 5.019, P = 0.056).

To check for the effect of obesity, we divided our biopsy population according to the presence of obesity (BMI ≥25 kg/m^2^) and undertook multivariate analysis. The DM+PC group was significantly associated with a higher rate of overall prostate cancer detection than the DM− group via biopsy, regardless of obesity status: non-obese men (OR = 2.715, P = 0.003) vs. obese men (OR = 2.344, P = 0.039). However, for the multivariate analysis of high-grade prostate cancer, there was no significant association.

## Discussion

In 2004, Bonovas S. and colleagues presented a meta-analysis about DM and the risk of prostate cancer. They involved 14 studies, published between 1971 and 2002, and suggested that there was strong evidence that diabetic people had a significant decrease in risk for developing prostate cancer [1]. Subsequently, in 2006, Kasper JS and colleagues announced similar results through their meta-analysis study, which involved 19 studies between 1971 and 2005 [2]. They strengthened the former contention.

However, because a considerable number of pre-PSA-era patients were included in those meta-analyses, strong doubts had been raised concerning the reliability of the results from these meta-analyses. In fact, several studies which targeted post-PSA-era patients have reported different results, and contrary to previous studies, Chan JM et al. analyzed of 6,722 men diagnosed as prostate cancer from 1989 to 2002 within CaPSURE data, and reported that any evidence of an inverse association between DM and prostate cancer risk was not observed [3]. On the other hand, Leitzmann MF et al. found out that a diabetic history was connected with a decreased risk of total prostate cancer among 33,088 men in the screening arm of the Prostate, Lung, Colorectal, and Ovarian (PLCO) Cancer Screening Trial in the late 2000s. However, through further analysis, they revealed that the association between diabetes and aggressive prostate cancer was suggestively positive for men who were lean (BMI <25 kg/m^2^) [4]. Moreia DM et al. retrospectively investigated 998 multiethnic men who underwent prostate biopsy between 2001 and 2009, and reported that DM was associated with a greater risk of high-grade disease in obese white men [5].

Several Asian papers joined in these conflicting results about the relationship between DM and prostate cancer. Li Q et al. examined the Ohsaki cohort followed from 1995 to 2003, in which 230 new cases of prostate cancer were identified among 22,458 Japanese men. They identified that after stratification based on the clinical stage of prostate cancer, diabetic patients showed a higher risk of advanced prostate cancer with a multivariate analysis [8]. In addition, Fukushima et al. retrospectively evaluated 2,038 men who had undergone prostate biopsy and concluded that DM was associated with more aggressive prostate cancer detection among obese Japanese patients with PSA level <10 ng/ml [9]. Hong et al. reviewed 3,925 men undergoing prostate biopsy and described that DM was significantly associated with a higher risk of overall prostate cancer detection and high-grade prostate cancer in a Korean biopsy population [7].

Meanwhile, some papers tried to investigate the role of glycemic control, as measured by HbA1c, on prostate cancer beyond the simple comparison of diabetic men versus non-diabetic men. Kim HS et al. identified 247 men from the Shared Equal Access Regional Cancer Hospital (SEARCH) database and detected men with higher HbA1c levels presented with more biologically aggressive prostate cancers at radical prostatectomy [10]. Hong et al. reported similar results. They reviewed 740 patients who underwent radical prostatectomy for clinically localized prostate cancer and announced that glycemic control, as represented by the HbA1c level, may be a useful preoperative predictor of aggressive tumor profiles among diabetic patients [11]. On the contrary, Onitilo AA et al. examined 9,486 type 2 DM patients and reported that prostate cancer risk was significantly higher with better glycemic control (HbA1c ≤7.0%) [12].

In our study, there were no significant results by the simple comparison with the existence of DM history. However, when we applied the classification according to the history of DM and the HbA1c level, we could find significant differences on the overall prostate detection rate between the DM− group and the DM+PC group. We divided the diabetic men into the DM+GC group and the DM+PC group by using the cut off value, HbA1c 6.5%, which is one of the current diagnostic criteria for DM [15]. Some papers have suggested that DM could have an influence on prostate cancer due to hormonal imbalance. The authors of those papers said that in diabetic patients there is a cause and effect relationship between hyperinsulinemia, low testosterone levels, and low PSA levels [16]–[19]. Supplementally, we expected that the effect of DM on prostate cancer wound be different depending upon the degree of glycemic control. In this study, when we analyzed the patients with negative biopsy, the PSA level was significantly different according to the history of DM, and the HbA1c level and the PSA level were lowest in the DM+PC group. Consequently, if the PSA level of diabetic patients would exceed the prostate biopsy indication despite the aforementioned hormonal imbalance, the prostate cancer risk would be increased paradoxically in DM patients, especially in the DM+PC group.

Meanwhile, we found that the DM+sPC group was significantly associated with a risk of high-grade prostate cancer in multivariate analysis. However, the significance level was marginal, and the number of patients in the DM+mPC group was small. As such, the finding was difficult to accept as a clear one. A number of studies have shown that low testosterone is related to high-grade prostate cancer and to a higher stage at presentation [20]–[24]. Although we did not present the low testosterone level in the DM+sPC group, if we assume that the testosterone level was lower, in the poor glycemic control group the results would be also explained within the concept of the hormonal imbalance.

We found significant difference in PSAD between groups in this study. When our analysis was performed with the exception of the 36 patients that showed excessive PSA, but with 50 or more of the total patients, PSA and PSAD were significantly lower than in the non-diabetic group, while prostate volume was no different. Several studies about the relationship between DM and prostate cancer have reported lower PSA in diabetic groups. These studies have presented the following reasons for such findings: 1) the impact of growth factors and hormone-related DM, 2) the effects of anti-diabetic drugs, and 3) intraprostatic vascular damage due to DM [25], [26]. Further research should clarify whether the changes in PSA or PSAD among different groups are found in general populations as well as biopsy populations.

Additionally, we could have found that abnormal DRE findings were more common in DM+PC group. However, there was no significant difference in the abnormal DRE findings when we re-divided the biopsy population into a DM group and a non-DM group (p = 0.097). Studies showing that the frequency of abnormal DRE findings were different depending on the degree of DM control are rare. In fact, ours may be the first such report. These findings should be verified through other studies in the future. Meanwhile, in our study, abnormal DRE findings were associated with overall prostate cancer detection, but were not a significant factor in high-grade prostate cancer detection.

In this study, from among the 1,368 patients suffering from biopsy complications, 16 men required hospitalization and additional antibiotic medication. The incidence of complications did not vary according to the presence or absence of DM. Carignan et al. have reported that in an analysis of 5,789 cases of prostate biopsies, DM was found to be one of the factors that can increase the risk of urinary sepsis as a biopsy complication [27].

Our study may be limited by its retrospective nature. Recently, there have been several reports about the effect of anti-diabetic drugs on prostate cancer. In particular, the relationship between metformin and prostate cancer is currently a hot issue [28]–[30]. Unfortunately, in this study, we could not assess the impact of the duration of DM and of DM medication use on the detection of prostate cancer. In addition, we did not assess other comorbidities and lifestyle factors, including diet and physical activity. However, as already mentioned, not many researchers have investigated the association between the level of glycemic control and prostate cancer. To our knowledge, our study is the first to recognize the significant association of HbA1c with prostate cancer in a biopsy population.

## Conclusions

Poor glycemic control of DM was associated with a higher risk of prostate cancer detection, including high-grade (Gleason score ≥7) disease, in the biopsy population. Further studies should be undertaken to elucidate the exact biological mechanism that exists between DM and prostate cancer.

## Ethical Standards

This study design and the use of patients' information stored in the hospital database were approved by the Institutional Review Board (IRB) at the Seoul Metropolitan Government - Seoul National University Boramae Medical Center. The approval number is 26-2013-84. We were given exemption from getting informed consents by the IRB because the present study is a retrospective study and personal identifiers were completely removed and the data were analyzed anonymously. Our study was conducted according to the ethical principles laid down in the 1964 Declaration of Helsinki and its later amendments.
